# Mesenchymal Stem Cells Restore Frataxin Expression and Increase Hydrogen Peroxide Scavenging Enzymes in Friedreich Ataxia Fibroblasts

**DOI:** 10.1371/journal.pone.0026098

**Published:** 2011-10-07

**Authors:** Kevin Kemp, Elizabeth Mallam, Kelly Hares, Jonathan Witherick, Neil Scolding, Alastair Wilkins

**Affiliations:** Multiple Sclerosis and Stem Cell Group, Institute of Clinical Neurosciences, School of Clinical Sciences, University of Bristol, Bristol, United Kingdom; University of South Florida, United States of America

## Abstract

Dramatic advances in recent decades in understanding the genetics of Friedreich ataxia (FRDA)—a GAA triplet expansion causing greatly reduced expression of the mitochondrial protein frataxin—have thus far yielded no therapeutic dividend, since there remain no effective treatments that prevent or even slow the inevitable progressive disability in affected individuals. Clinical interventions that restore frataxin expression are attractive therapeutic approaches, as, in theory, it may be possible to re-establish normal function in frataxin deficient cells if frataxin levels are increased above a specific threshold. With this in mind several drugs and cytokines have been tested for their ability to increase frataxin levels. Cell transplantation strategies may provide an alternative approach to this therapeutic aim, and may also offer more widespread cellular protective roles in FRDA. Here we show a direct link between frataxin expression in fibroblasts derived from FRDA patients with both decreased expression of hydrogen peroxide scavenging enzymes and increased sensitivity to hydrogen peroxide-mediated toxicity. We demonstrate that normal human mesenchymal stem cells (MSCs) induce both an increase in frataxin gene and protein expression in FRDA fibroblasts via secretion of soluble factors. Finally, we show that exposure to factors produced by human MSCs increases resistance to hydrogen peroxide-mediated toxicity in FRDA fibroblasts through, at least in part, restoring the expression of the hydrogen peroxide scavenging enzymes catalase and glutathione peroxidase 1. These findings suggest, for the first time, that stem cells may increase frataxin levels in FRDA and transplantation of MSCs may offer an effective treatment for these patients.

## Introduction

Friedreich ataxia (FRDA) is the commonest autosomal recessive ataxic condition, affecting around 1 in 50,000 of the population [1]. FRDA is caused by a homozygous GAA repeat expansion mutation within intron 1 of the *FXN* gene (formerly FRDA gene; [2]). GAA triplet expansion leads to greatly reduced expression of frataxin, a mitochondrial protein the functions of which include iron chaperoning in iron-sulphur clusters and heme biosynthesis; maintenance of anti-oxidant defences; and iron detoxification [3], [4]. The correlation between reduced frataxin levels (longer GAA expansions) and earlier onset neurological disease implies a role for frataxin in maintenance and protection of neurons [5]. The precise subsequent mechanisms of cell death and neurodegeneration remain the subject of active research, an increasing consensus suggests that oxidative damage plays a key role [6], [7].

Currently there are no effective treatments to prevent the progression of FRDA. Major therapeutic strategies in FRDA include development of agents to protect against oxidative damage and mitochondrial respiratory chain defects or agents that increase cellular frataxin expression, since cells lacking frataxin can be rescued through frataxin expression [8]. With this in mind several drugs and cytokines have been tested for their ability to increase frataxin levels in both *in vitro* and *in vivo* [9]–[14]. We hypothesised that cell replacement therapies could potentially address both these therapeutic aims, influencing frataxin expression and offering more widespread cellular protective roles in FRDA. A large body of research suggests that transplantation of mesenchymal stem cells (MSCs) has therapeutic potential for neurodegenerative disorders through a multitude of different mechanisms. These include replacement of lost cells by differentiation into functional tissue, modulation of the immune system to prevent further degeneration, and/or provision of trophic support for the diseased cellular system [15]. Increasing evidence suggests that the major mechanistic protective role of MSCs is their capacity to promote an environment conducive to cellular protection and to support cell survival through the secretion of a diverse range of potentially protective factors, including those with anti-oxidant properties [15]–[20].

Administration of MSCs is an attractive therapeutic option for FRDA for several reasons: cells are easily isolated from various anatomical sources, have a versatile growth and differentiation potential, and, in addition, the immunosuppressive properties of MSCs make it probable that allogeneic as well as autologous cell therapy could be considered. If human MSC transplantation is to become a viable therapeutic approach for FRDA, a precise understanding of the effects of MSCs and their secreted factors on cells derived from FRDA patients is required.

Here we have performed a series of experiments showing that human MSCs increase both frataxin gene and protein expression in fibroblasts derived from patients with FRDA via secretion of soluble factors. We show a direct link between frataxin expression in fibroblasts derived from FRDA patients with both decreased expression of hydrogen peroxide scavenging enzymes and increased sensitivity to hydrogen peroxide-mediated toxicity. Furthermore, we have demonstrated that exposure to factors produced by MSCs increase resistance to hydrogen peroxide mediated toxicity in these fibroblasts through, at least in part, restoring the expression of the hydrogen peroxide scavenging enzymes catalase and glutathione peroxidase 1. These findings suggest that MSCs may have potential as therapeutic agents for FRDA and, in time, transplantation of bone marrow-derived MSCs may offer an effective cellular therapy in these patients.

## Materials and Methods

### Patients and fibroblast culture

Primary fibroblast cells, derived from skin biopsies of Caucasian FRDA and control patients, were obtained from the Coreill cell repositories (Coreill Institute for medical research, New Jersey, USA). Fibroblasts were grown in DMEM with 10% foetal calf serum (FCS) (StemCell Technologies, London, UK), and 100 U/ml penicillin and 100 µg/ml streptomycin (Sigma-Aldrich, Gillingham, UK). All experiments were on fibroblast cultures between 8–12 passages.

### Transfection of fibroblasts with GFP-tagged *FXN*

Transfection of fibroblasts was carried out using Amaxa Nucleofector kits for primary cells (Lonza, Basel, Switzerland). Prior to nucleofection, confluent fibroblast cultures trypsinized with trypsin-EDTA. Proteolysis by trypsin was stopped with the addition of DMEM supplemented with 10% FCS. 3×10^5^ cells were then transfected with 2.0 µg GFP-tagged ORF clone of human frataxin (*FXN*) (Origene, Maryland, USA) according to manufactures instructions. The nucleofector programme U-23 was chosen for high transfection efficiency. Cells were then re-suspend in 2ml DMEM/10% FCS and cultured within one well of a 6-well plate. Fluorescence microscopy was used to visualise GFP-tagged frataxin within *FXN* transfected fibroblasts. For a mock-treated control, distilled water was used to replace the vector expression system.

### Establishment of mesenchymal stem cell cultures from healthy control patients

Bone marrow samples were obtained by an orthopaedic surgeon at Southmead Hospital, Bristol, UK with informed written consent and hospital ethic committee approval by the North Bristol NHS trust research ethics committee. Bone marrow was taken at the time of total hip replacement surgery from the femoral shaft and placed into a sterile 50ml tube containing 1000 I.U heparin. Patients with a history of malignancy, immune disorders or rheumatoid arthritis were excluded from the study. Femoral shaft bone marrow donors were healthy apart from osteoarthritis, and were not receiving drugs known to be associated with myelosuppression or bone-marrow failure.

Femoral shaft marrow samples were broken up with a scalpel and washed with Dulbecco's Modified Eagles Medium (DMEM) (Sigma-Aldrich, Gillingham, UK) until remaining material (bone) looked white at the bottom of the 50 ml tube. All washings were pipetted into a new 50 ml tube and kept for centrifugation. The suspension was centrifuged and re-suspended in DMEM and overlaid onto an equal volume of Lymphoprep (Axis-Shield, Dundee, UK; density 1.077+/−0.001 g/ml) and centrifuged at 600 g for 35 minutes at room temperature to separate the mononuclear cells (MNC) from neutrophils and red cells. The MNC layer was harvested and washed twice in DMEM.

### MSC culture

Isolated MNCs were centrifuged and re-suspended in MSC medium consisting of DMEM with 10% foetal calf serum (FCS) selected for the growth of MSCs (StemCell Technologies, London, UK), and 100 U/ml penicillin and 100 µg/ml streptomycin (Sigma-Aldrich, Gillingham, UK). Vented flasks (25 cm^2^) containing 10 ml of MSC medium were seeded with 1×10^7^ cells for primary culture. Flasks were incubated at 37°C in a humidified atmosphere containing 5% CO_2_ and fed every week with MSC medium by half medium exchange to remove non-adherent hematopoietic cells until the adherent fibroblast-like MSCs reached approximately 70% confluence. On reaching confluence the adherent cells were re-suspended using 0.25% trypsin (Sigma-Aldrich, Gillingham, UK) and re-seeded at 2.25×10^5^ cells per (75 cm^2^) flask into first passage. Cultures were then incubated, fed every week with MSC medium by half medium exchange, and again trypsinised, a cell count taken and re-seeded at 2.25×10^5^ cells per flask (75 cm^2^).

### MSC characterization

MSCs harvested from femoral shaft bone marrow were cultured and characterised according to previous published reports from our laboratory [16]–[18], [21], [22]. Briefly, MSC cultures were characterised at third passage using anti-CD105, anti-CD45 (eBioscience, California, USA), anti-CD166 and with anti-CD44 (Serotec, Oxford, UK). MSCs were also differentiated down the adipogenic, osteoblastic and chondrogenic lineages.

### Preparation of mesenchymal stem cell conditioned medium

Confluent MSCs, at third passage, were washed in DMEM and cultured for 24hours in minimal base medium (consisting of Dulbecco's modified eagles medium supplemented with insulin-free Sato (containing 100 µg/ml bovine serum albumin, 100 µg/ml transferrin, 0.06 µg/ml progesterone, 16 µg/ml putrescine, 0.04 µg/ml selenite, 0.04 µg/ml thyroxine, 0.04 µg/ml tri-iodothryonine)[18]. Medium was then removed and stored at −80°C prior to being used within culture experiments. MSC-conditioned medium in this study was prepared from at least 6 independent MSC samples.

### Erythropoietin enzyme linked immunosorbent assay (ELISA)

Mesenchymal stem cell-conditioned medium, derived from MSCs at varying passages, was analyzed by ELISA using the Human Erythropoietin ELISA kit (Abnova, Heidelberg, Germany) according to the manufacturer's instructions. All samples were analyzed in triplicate.

### Real-time Polymerase Chain Reaction (PCR)

Fibroblast cultures were washed in DMEM, trypsinised and resuspended in PBS. RNA was extracted and cDNA produced using the Taqman gene expression cells-Ct-kit (Applied Biosystems, Paisley, UK) according to the manufacturer's instructions. 10^5^ cells were added to lysis solution plus DNase (5 minutes) before the addition of stop solution (2 minutes). All RNA samples were quantified using a Qubit Fluorometer and Quant-iT RNA assay kit (Invitrogen, Paisley, UK) according to manufacturers' instructions to ensure equal loading of RNA samples. Samples were stored at −80°C prior to use. To synthesize cDNA, 100 ng of extracted RNA was added to the reverse transcription buffer and RT enzyme mix, placed in a thermal cycler, and incubated at 37°C for 1 hour and 95°C for 5 minutes. RT-PCR was performed using the StepOnePlus Real-Time PCR System (Applied Biosystems, Paisley, UK) with Assay-on-demand Gene Expression Products for *FXN* (Taqman MGB probe, FAM dye-labelled, Applied Biosystems, Paisley, UK) or 18S rRNA (Taqman MGB probe, VIC dye-labelled, Applied Biosystems, Paisley, UK) using 10 ng of cDNA in a total volume of 20 µl. Reactions were run at 50°C for 2 minutes; 95°C for 10 minutes; and 40 cycles of 95°C for 15 seconds and 60°C for 1 minute. All samples were analyzed in triplicate. The relative gene expression (RQ value) of *FXN* was calculated using the 2^-ΔΔCt^ method, and the mean taken for each group. 18S rRNA was used as the reference ‘housekeeping’ gene.

### Immunoblotting

Fibroblasts cultured in a 6-well plate were lysed using Beadlyte cell signalling universal lysis buffer (Millipore, Watford, UK). The Qubit Fluorometer and Quant-iT protein assay kit (Invitrogen, Paisley, UK) was then used to quantify the concentration of total protein within each cell lysate sample according to manufacturers' instructions to ensure equal loading of cell lysates. Lysates were heated to 95°C for five minutes with Laemmli 2x sample buffer (Invitrogen, Paisley, UK) and run on Tris-HCl 10–20% ready gels (Bio-Rad, Hemel Hempstead, UK). After transfer to a nitrocellulose membrane (Bio-Rad, Hemel Hempstead, UK) and blocking in Tris-buffered saline/5% bovine serum albumin, membranes were incubated overnight in primary antibody at 4°C (in Tris-buffered saline/5% bovine serum albumin). Antibodies used were mouse anti-human frataxin (this antibody recognizes a band migrating at approximately 18 kDa corresponding to the mature Frataxin protein isoform (aa 56–210)) (1∶10000; Millipore, UK), mouse anti-GAPDH (1∶5000; Abcam, Cambridge, UK), rabbit anti-catalase (1∶15000; Abcam, UK), rabbit anti-glutathione peroxidase 1 (GPX1) (1∶5000; Abcam, Cambridge, UK). Immunoreactivity was detected using secondary anti-rabbit or anti-mouse horseradish peroxidase conjugated antibodies (Abcam, Cambridge, UK) (in Tris-buffered saline/5% bovine serum albumin) and specific protein expression patterns were visualized by chemiluminescence using an Amersham ECL Plus Western Blotting Detection System (GE Healthcare, Little Chalfont, UK). Densitometic analysis of western blot bands was performed using ImageJ software (NIH, Maryland, USA).

### Frataxin lateral flow dipstick immunoassay

Frataxin levels in fibroblasts-derived from patients with FRDA were measured using MitoSciences frataxin dipstick (MSF31) (Mitosciences, Oregon, USA) assays according to manufacturers' instructions. Briefly, 10 µg of fibroblast protein in 25 µL of extraction buffer was mixed with 25 µL blocking buffer and added to individual wells on a 96-well plate with gold-conjugated monoclonal antibody at the bottom of each well. Dipsticks were then inserted into the wells and frataxin within the each sample was immunocaptured onto designated capture zones on the dipstick. Densitometic analysis of capture zones on developed dipsticks were quantified using ImageJ software (NIH, Maryland, USA).

### Assessment of H_2_O_2_ cytotoxicity

Confluent fibroblast cultures were washed in DMEM, trypsinised and cultured in 96-well plates (10,000 cells per well) in DMEM/10%FCS. At 24 hours *in vitro* culture, cells were exposed to experimental conditions. Medium was removed from all wells and cells were washed twice in DMEM. Minimal medium and/or MSC conditioned medium was added to appropriate wells with/without the addition of 3-AminoTriazole (10mM, Sigma, Gillingham, UK). After a further 24 hours media was removed from all wells and replaced with minimal medium containing hydrogen peroxide (600 µM) (Sigma-Aldrich, Gillingham, UK) with/without the addition of 3-AminoTriazole (10mM, Sigma, Gillingham, UK), catalase from human erythrocytes (10 U/ml, Sigma, Gillingham, UK), glutathione peroxidase from human erythrocytes (10U/ml, Sigma, Gillingham, UK) (An initial dose response curve to determine the optimal hydrogen peroxide concentration (600 µM) for the given timed cell survival experiment (data not shown)). Hydrogen peroxide levels were detectable in the media for approximately 5 hours, therefore at 6 hours post addition of hydrogen peroxide, evaluation of fibroblast cell survival was carried out using the 3-(4,5-Dimethylthiazol-2-yl)-2,5-diphenyltetrazolium bromide (MTT) cell viability assay (96-well plates). For assessment of hydrogen peroxide cytotoxicity in *FXN*-transfected cells, fibroblasts were cultured in DMEM/10%FCS for a total of 48 hours after seeding into wells prior to varying concentrations hydrogen peroxide for 6 hours as above.

#### MTT cell viability assay

Briefly, the supernatant was removed and replaced with HBSS/10%FCS containing 1 mg/ml MTT (Sigma-Aldrich, Gillingham, UK). Cells were then incubated for 1 hour at 37°C and subsequently the HBSS/10%FCS/MTT solution was removed by inverting the plate and the plate was left to dry. DMSO (Sigma-Aldrich, Gillingham, UK) was then added to each well and the absorbance of the solution in each well read in a plate reader at 540 nm. Cell survival was assessed as a ratio of the amount of formazan production relative to the respective control. In all cases, control cultures grown throughout the experimental period in base ‘minimal’ medium were analyzed and values for the experimental conditions divided by this value, in order to standardize results between experiments.

### Statistical analysis

Statistical comparisons were analyzed using one way ANOVA with *post hoc* Bonferroni testing between groups where appropriate. Paired or unpaired t-tests were used for analysis of normally distributed data. Values are expressed as the mean +/− SEM from at least three independent experiments, taking p<0.05 to represent statistical significance.

## Results

Primary fibroblast cultures derived from skin biopsies of FRDA and healthy control patients were established (see Table 1). Fibroblast cultures were morphologically homogenous and characterized by their spindle-like appearance. To investigate the effects MSCs have on frataxin expression in fibroblasts derived from patients with FRDA, patient fibroblasts were exposed to MSC conditioned medium *in vitro* and frataxin expression investigated using western blotting and RT-PCR techniques.

**Table 1:** Subject description of Friedreich ataxia patients and age matched healthy controls.

Patient	Sex	Age	GAA expansions	Phenotype
FRDA1	F	36	330 and 380 repeats	Clinically affected; spinal-cerebral degeneration with cardiomyopathy
FRDA2	F	13	780 and 780 repeats	Clinically affected; limb and gait ataxia; scoliosis; proprioceptive sensory loss
FRDA3	M	30	541 and 420 repeats	Clinically affected; ataxia; cardiomyopathy; mild peripheral neuropathy
CON1	F	36	n/a	normal control
CON2	F	16	n/a	normal control
CON3	M	30	n/a	normal control

### Frataxin expression in human fibroblasts derived from Friedreich ataxia and healthy control patients

**Figure 1:**
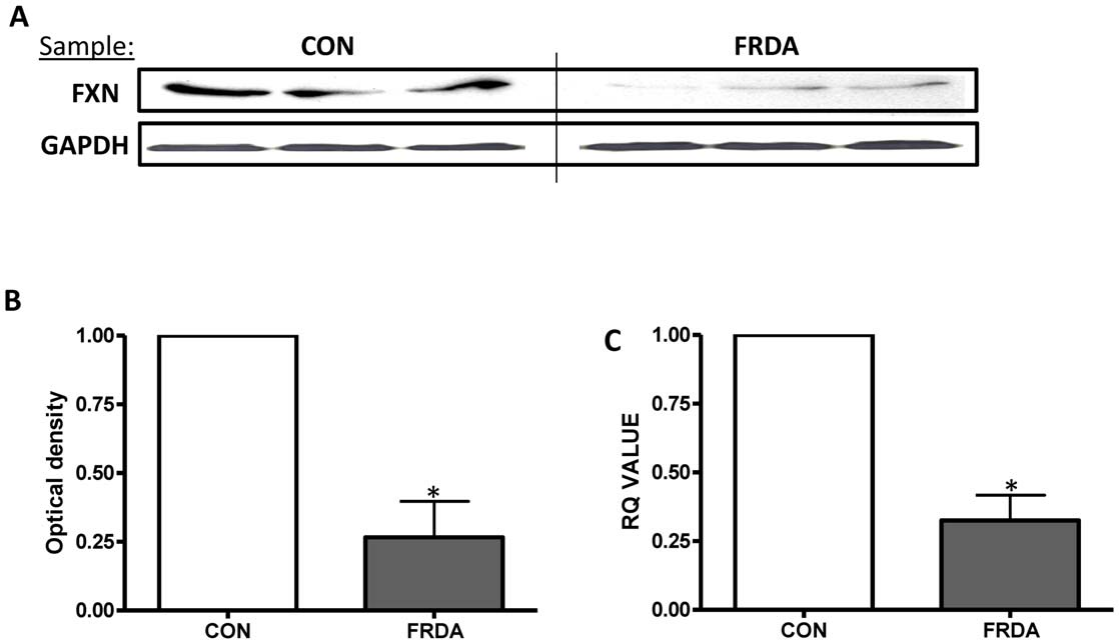
Fibroblasts derived from patients with Friedreich ataxia have low frataxin expression. (A) Immunoblotting of human frataxin in FRDA and control (CON) fibroblasts. Upper panels correspond to frataxin (FXN); lower panel corresponds to the loading control GAPDH. (B) Densitometic analysis of frataxin expression of western blot bands. Data are given using arbitrary units of integrated density. (C) The relative frataxin mRNA expression in control and FRDA fibroblasts . Results are expressed as the mean +/− (SEM). (*p<0.05, comparing test condition to control; n = 3 independent experiments).

Western blot analysis was used to detect the cellular protein expression of frataxin, and to verify the characteristic decrease in frataxin expression within cells derived from patients with FRDA. Detection using a anti-human frataxin (exon 4) IgG2 antibody revealed a frataxin protein band at approximately 18kDa for both healthy control and FRDA cell lysates which corresponds to the predicted molecular weight of frataxin (Figure 1A). Image analysis using ImageJ software calculated that frataxin expression in fibroblasts derived from patients with FRDA have a significantly lower in expression of the 18kDa band (p<0.05), with an approximate 75% decrease in frataxin expression (Figure 1B). GAPDH expression was used to ensure equal loading of cell lysates. Frataxin expression by fibroblasts derived from both healthy control and FRDA patients was also investigated at the genomic level using RT-PCR. Frataxin mRNA levels differed significantly between healthy control and FRDA fibroblasts (p<0.05). Calibration of frataxin mRNA levels with reference to 18S rRNA revealed that fibroblasts derived from patients with FRDA had also an approximate 75% decrease in frataxin mRNA expression, under normal culture conditions, when compared to the control (Figure 1C).

### Mesenchymal stem cell conditioned medium significantly increases Frataxin protein expression

**Figure 2:**
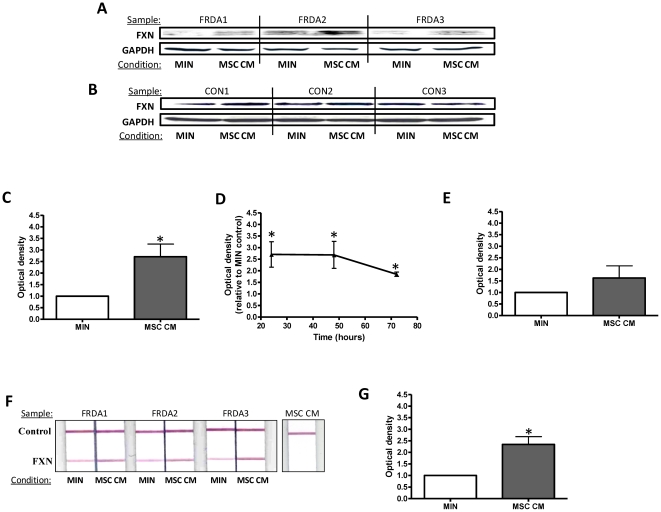
Mesenchymal stem cell conditioned medium increases frataxin protein expression. Immunoblotting of human frataxin in (A) FRDA fibroblasts and (B) control fibroblasts after exposure to minimal medium (MIN) or MSC conditioned medium (MSC CM) for 24 hours. Upper panels correspond to frataxin (FXN); lower panel corresponds to the loading control GAPDH. Western blot densitometic analysis of frataxin expression in fibroblasts derived from patients with Friedreich ataxia at (C) 24 hours and (D) over a 72 hour period. (E) Western blot densitometic analysis of frataxin expression in fibroblasts derived from healthy controls. (F) The dipstick immunoassay of human frataxin in FRDA fibroblasts (or MSC CM alone) after exposure to minimal medium (MIN) or MSC CM for 24 hours. Upper panels correspond to internal control; lower panel corresponds to human frataxin (FXN). (G) The dipstick immunoassay densitometic analysis of frataxin expression in fibroblasts derived from patients with Friedreich ataxia. Data are given using arbitrary units of integrated density. Results are expressed as the mean +/− (SEM). (*p<0.05, comparing test condition to control; n = 3 independent experiments).

Having shown a significantly reduced frataxin expression in fibroblasts derived from patients with FRDA, we next wished to determine the effects of MSC-conditioned medium on frataxin expression by these cells. Fibroblast cells were cultured for 24 hours before exposure to experimental conditions. Using immunoblotting techniques, after 24 hours post exposure to MSC-conditioned medium, frataxin expression in fibroblasts derived from patients with FRDA (Figure 2A) and healthy controls (Figure 2B) was detected. Image analysis using ImageJ software calculated that in the presence of MSC-conditioned medium frataxin expression in fibroblasts derived from patients with FRDA was significantly up-regulated by 2.7-fold after 24 hours exposure when compared to base minimal media alone (p<0.05) (Figure 2C). This increase in frataxin expression was evident for up to 72hours post exposure to MSC-conditioned medium (Figure 2D) (p<0.05). No significant differences in frataxin expression were evident in fibroblasts derived from healthy controls after 24 hours exposure to MSC-conditioned medium when compared to base minimal media alone (p>0.05) (Figure 2E). GAPDH expression was used to ensure equal loading of cell lysates.

A frataxin lateral flow dipstick immunoassay was also used to investigate the MSC-conditioned medium induced increase in frataxin expression in fibroblast derived from patients with FRDA. After 24 hours post exposure to MSC-conditioned medium, frataxin expression in fibroblasts derived from patients with FRDA was detected (Figure 2F). Image analysis using ImageJ software calculated that in the presence of MSC-conditioned medium frataxin expression in fibroblasts derived from patients with FRDA by was significantly up-regulated by approximately 2.4-fold after 24 hours exposure when compared to base minimal media alone (p<0.05) (Figure 2G). It was also shown that there was no frataxin present within the MSC-conditioned medium sample alone (Figure 2F).

### Mesenchymal stem cell conditioned medium significantly increases Frataxin gene expression

**Figure 3:**
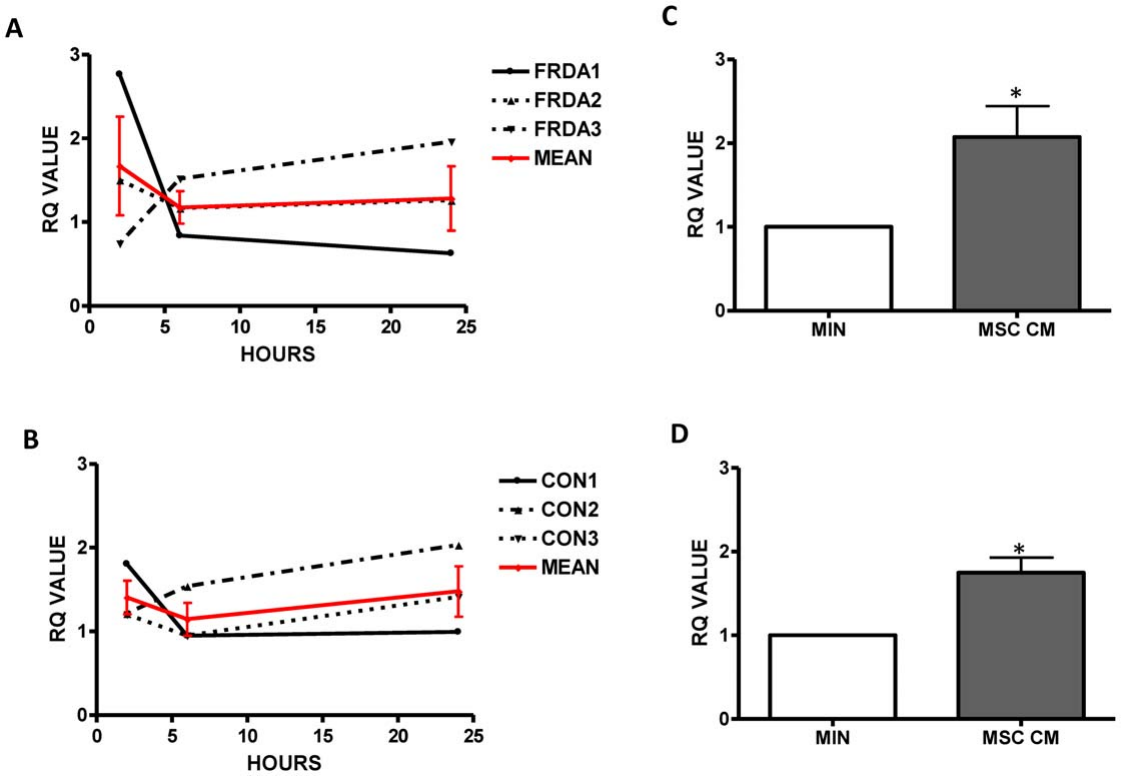
Mesenchymal stem cell conditioned medium increases frataxin gene expression. The relative frataxin mRNA expression in (A) FRDA and (B) control fibroblasts after exposure to minimal medium (MIN) or MSC conditioned medium (MSC CM) for 2, 6 and 24 hours. The mean maximal relative frataxin mRNA expression in (C) FRDA and (D) control fibroblasts evident throughout the 24 hour exposure to minimal medium (MIN) or MSC CM. Results are expressed as the mean +/− (SEM). (*p<0.05, comparing test condition to control; n = 3 independent experiments).

To gain a further understanding into the mechanisms by which MSC-conditioned medium promotes an increase in frataxin expression, we investigated frataxin expression at the genomic level using RT-PCR. At 2, 6 and 24 hours frataxin mRNA levels from both FRDA patients (Figure 3A) and healthy controls (Figure 3B) were investigated post exposure to either MSC-conditioned medium or minimal media alone. Calibration of frataxin mRNA levels was adjusted with reference to 18S rRNA. At each time point, no significant differences in frataxin mRNA expression levels were evident between cells exposed to either MSC-conditioned medium or minimal media alone (p>0.05). Analysing the maximal relative frataxin mRNA expression observed over the 24 hour period, revealed that exposure to MSC-conditioned medium resulted in an approximate 2-fold increase in frataxin mRNA expression in fibroblasts derived from both patients with FRDA (Figure 3C) and healthy controls (Figure 3D), when compared to exposure to minimal medium alone (p<0.05).

### Mesenchymal stem cells increase frataxin expression through a mechanism independent of erythropoietin secretion

To determine if MSCs secrete erythropoietin, we performed an erythropoietin ELISA on four different MSC-conditioned medium samples. Using this quantitative method, no detectable amount of erythropoietin was observed in any of the samples tested (data not shown).

### Mesenchymal stem cell conditioned medium significantly increases both catalase and glutathione peroxidase 1 expression

**Figure 4:**
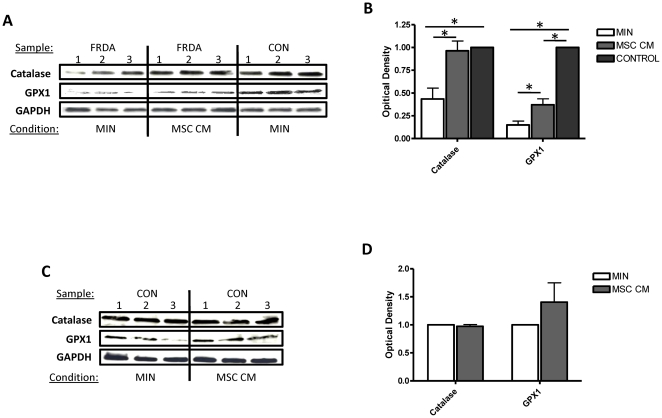
Mesenchymal stem cell conditioned medium increases both catalase and glutathione peroxidase 1 expression in fibroblasts-derived from FRDA patients. Immunoblotting of human catalase, glutathione peroxidase 1 (GPX1) and loading control GAPDH in (A) FRDA/control fibroblasts and (C) control fibroblasts after exposure to minimal medium (MIN) or MSC conditioned medium (MSC CM) for 24 hours. Western blot densitometic analysis of catalase and GPX1expression in fibroblasts derived from (B) patients with FRDA/controls and (D) control fibroblasts at 24 hours. Data are given using arbitrary units of integrated density. Results are expressed as the mean +/− (SEM). (*p<0.05, comparing test condition to control; n = 3 independent experiments).

We next determined the effects of MSC-conditioned medium on expression of the hydrogen peroxide scavenging enzymes, catalase and glutathione peroxidase 1, in fibroblasts derived from FRDA patients and healthy controls. Fibroblast cells were cultured for 24 hours before exposure to experimental conditions. Using immunoblotting techniques and image analysis using ImageJ software, it was calculated that both catalase and glutathione peroxidase 1 expression in fibroblasts derived from patients with FRDA was significantly lower in expression when compared to the age matched healthy controls (Figure 4A & Figure 4B) (p<0.05). After 24 hours exposure to MSC-conditioned medium both catalase and glutathione peroxidase 1 expression in fibroblasts derived from patients with FRDA was significantly up-regulated by approximately 2-fold when compared to base minimal media alone (p<0.05) (Figure 4A & Figure 4B). No significant differences in catalase and glutathione peroxidase 1 expression were evident in fibroblasts derived from healthy controls after 24 hours exposure to MSC-conditioned medium when compared to base minimal media alone (p>0.05) (Figure 4C & Figure 4D). GAPDH expression was used to ensure equal loading of cell lysates.

#### Increased frataxin expression in *FXN* transfected FRDA fibroblasts increases catalase expression

**Figure 5:**
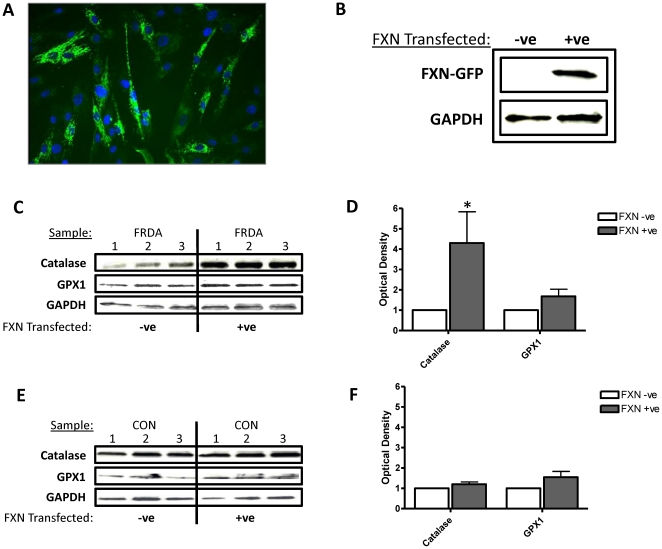
Enhanced frataxin expression in *FXN*-transfected FRDA fibroblasts increases catalase expression. (A) FRDA fibroblast culture transfected with a GFP-tagged *FXN* gene. (B) Immunoblotting of human frataxin in GFP-tagged frataxin transfected (right) and untransfected (left) FRDA fibroblasts. Upper panels correspond to frataxin; lower panel corresponds to the loading control GAPDH. Immunoblotting of human catalase, glutathione peroxidase 1 (GPX1) and loading control GAPDH in (C) FRDA fibroblasts and (E) control fibroblasts after transfection with the GFP-tagged *FXN* gene. Western blot densitometic analysis of catalase and GPX1expression in fibroblasts derived from (D) patients with FRDA and (F) control fibroblasts at 24 hours. Data are given using arbitrary units of integrated density. Results are expressed as the mean +/− (SEM). (*p<0.05, comparing test condition to control; n = 3 independent experiments).

Nucleofection efficiently delivered GFP-tagged *FXN* with approximately 76% (SE +/− 2) of the fibroblasts expressing of GFP 48 hours following nucleofection (Figure 5A). An increase in frataxin protein expression 48 hours post nucleofection was also confirmed using immunoblotting techniques. Detection using anti-frataxin antibody revealed a protein band at approximately 45kDa for transfected cell lysates which corresponds to the expected weight of the GFP-tagged frataxin protein. This band was absent from non-DNA transfected controls (Figure 5B). GAPDH expression was used to ensure equal loading of cell lysates.

We examined whether there was a direct link between frataxin expression and the expression of both catalase and glutathione peroxidase 1 in fibroblasts derived from patients with FRDA and healthy controls. Using immunoblotting techniques and image analysis using ImageJ software, it was calculated that *FXN*-transfection in fibroblasts-derived from FRDA patients led to a 4-fold increase in catalase expression (p<0.05), no significant increase in glutathione peroxidase 1 expression was evident (p>0.05) (Figure 5C & Figure 5D). *FXN*-transfection in fibroblasts-derived from healthy controls resulted in no significant differences in catalase or glutathione peroxidase 1 expression (p>0.05) (Figure 5E & Figure 5F). GAPDH expression was used to ensure equal loading of cell lysates.

### Mesenchymal stem cell conditioned medium or *FXN*-transfection increases resistance to hydrogen peroxide mediated toxicity in fibroblasts derived from Friedreich ataxia patients

**Figure 6:**
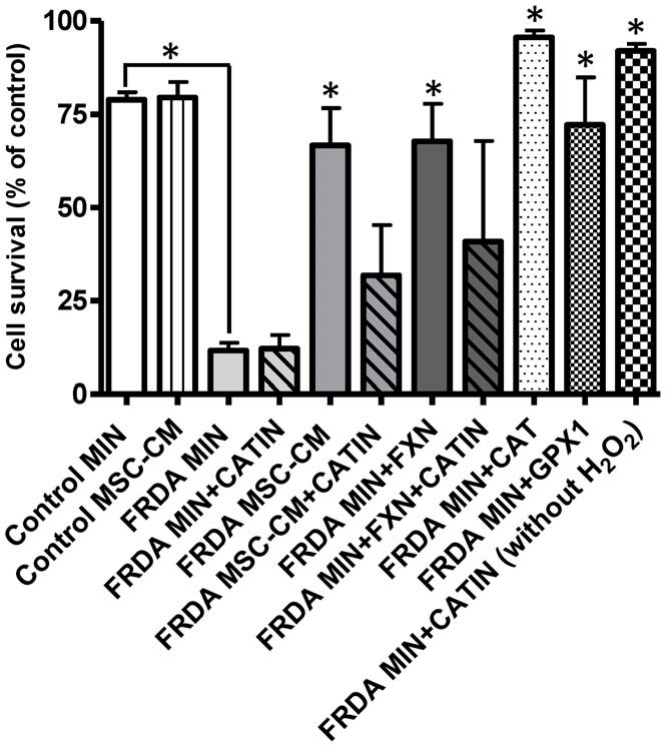
Mesenchymal stem cell conditioned medium or *FXN*-transfection increases resistance to hydrogen peroxide mediated toxicity in fibroblasts derived from FRDA patients. The effect of hydrogen peroxide (600 µM) on FRDA and control fibroblast cell survival *in vitro* post exposure to minimal medium (MIN) or MSC-conditioned medium (MSC CM) for 24hours, with/without the addition of the catalase inhibitor 3-AminoTriazole (CATIN), catalase (CAT) and glutathione peroxidase (GPX1). FXN indicates fibroblasts have been transfected with the GFP-tagged *FXN* gene. Cell survival was assesed using the MTT cell viability assay (MTT). Cell survival is expressed as a percentage of cell survival compared to cells grown in minimal medium alone. Results are expressed as the mean +/− (SEM). (*p<0.05, comparing test condition to control; n = 3 independent experiments).

Next we examined the toxicity of hydrogen peroxide to both fibroblasts derived from healthy controls and patients with FRDA using a MTT cell viability assay. Initial dose response curves were used to determine the optimal hydrogen peroxide concentrations. Cells were either incubated with minimal medium or MSC-conditioned medium for 24hours prior to insult with/without the addition of the catalase inhibitor 3-AminoTriazole. Firstly it was shown that fibroblasts from healthy controls were significantly more resistant to hydrogen peroxide mediated cellular toxicity when compared to fibroblasts derived from patients with FRDA when under normal control conditions (p<0.05) (Figure 6). Following this it was shown that exposure to MSC-conditioned medium or *FXN*-transfection in fibroblasts-derived from patients with FRDA resulted in a significant increase in cell survival when compared to cells exposed to minimal medium alone at identical concentrations of hydrogen peroxide (p<0.05) (Figure 6). The addition of the catalase inhibitor 3-AminoTriazole abrogated this increase in cell survival in the cells exposed to MSC-conditioned medium. The addition of 3-AminoTriazole alone without hydrogen peroxide exposure, to fibroblasts derived from FRDA patients, had no significant effect on cell survival. No significant differences in cell survival after hydrogen peroxide treatment were evident in fibroblasts derived from healthy controls, post exposure MSC-conditioned media.

Both the addition of catalase or glutathione peroxidase (derived from human erythrocytes) at the time of hydrogen peroxide exposure, led to an increase in FRDA fibroblast cell survival when compared to hydrogen peroxide alone (p<0.05) (Figure 6).

## Discussion

Friedreich ataxia is an incurable, progressive neurodegenerative condition characterized by multiple symptoms including gait and limb ataxia, dysarthria and an array of systemic complications including diabetes mellitus, scoliosis and hypertrophic cardiomyopathy which is the principle cause of death [23]. FRDA is typically caused by a homozygous GAA repeat expansion within the frataxin gene, resulting in a reduced expression of the frataxin protein. However, a small proportion of patients are heterozygous for the expanded allele with the addition of a mutation in the second allele leading to a premature stop codon [2], [24]. The amount of frataxin gene and protein expression in clinically symptomatic patients varies from approximately 5–35% of normal levels.

Heterozygous carriers of the GAA expansion, who are asymptomatic, have approximately 50% of normal frataxin expression [25], [26]. Thus, clinical interventions that are able to increase the amount of the frataxin protein are attractive therapeutic approaches, as in theory, it may be possible to re-establish normal function in frataxin defective cells by increasing frataxin levels above a specific threshold. It has been demonstrated frataxin knockout cells can be rescued through the insertion of frataxin using gene therapy techniques [8]. At present there are several strategies for the treatment of specific symptoms of frataxin deficiency including agents that protect against oxidative damage and mitochondrial respiratory chain defects (Idebenone, Pioglitazone and Deferiprone) [27]–[29]. However, several agents that are able to increase frataxin expression (recombinant erythropoietin and histone deacetylase enzyme inhibitors (HDACi)) are also being tested [9]–[14].

In this study, we have performed a series of experiments investigating the effects human MSCs have on both frataxin gene and protein expression in fibroblasts derived from patients with FRDA and healthy controls. We show that human MSCs, via the secretion of soluble factors, are able to increase frataxin mRNA expression promoting a prolonged increase in frataxin protein expression in fibroblasts derived from patients with FRDA. Increases in frataxin mRNA expression over a 24 hour exposure to MSC-condition medium were also evident within our healthy control group although no significant differences in frataxin protein expression were observed. This may however be associated with the relatively low sample numbers in this group. It is believed that GAA repeats in FRDA patients block transcription via several proposed mechanisms including impeding the progress of RNA polymerase [30] and the induction of an inaccessible heterochromatin structure [31]. The different GAA expansion lengths in the FRDA patients could therefore explain the large variation in the induction time of frataxin mRNA synthesis post exposure to MSC-conditioned medium. Indeed, if any increase in frataxin mRNA is observed a prolonged increase of mature frataxin protein would be expected to follow due to the half-life of mature frataxin being approximately two days [32]. The maximal frataxin mRNA expression of each sample post exposure to MSC-conditioned medium resulted in a 2-mean fold increase in frataxin mRNA expression in fibroblasts derived from patients with FRDA. This correlated well with the prolonged increase in frataxin protein expression observed in cells derived from FRDA patients. Similar studies using HDAC inhibitors and erythropoietin have reported increases in frataxin protein expression in primary cells derived from patients with FRDA *in vitro* [9], [10], [13], [14], with studies showing increases in frataxin protein with and without corresponding increases in frataxin mRNA [9], [13]. Increases in frataxin protein expression have also been confirmed using recombinant erythropoietin in clinical studies in FRDA patients [11], [33], [34]. To determine if MSCs secrete erythropoietin, thus a possible mechanism by which they promote an increase in frataxin expression, we analyzed several different MSC-conditioned medium samples using an erythropoietin ELISA. Using this method, no detectable amount of erythropoietin was present, therefore indicating MSCs increase frataxin expression through a mechanism independent of erythropoietin secretion.

Oxidative damage and inhibition of mitochondrial function may be key determinants of cellular damage and the pathogenesis of FRDA. Cells deficient of frataxin are shown to have a greater sensitivity to oxidative stress [35]–[38], and are also shown to have an impaired ability to recruit antioxidant defences against an endogenous oxidative stress [39]. Increased sensitivity of FRDA fibroblasts to hydrogen peroxide has been previously reported [40]–[43]. As hydrogen peroxide reacts slowly with biological molecules, hydrogen peroxide mediated cellular toxicity mainly occurs through labile (chelatable) iron ions, which catalyze the formation of the hydroxyl radical from hydrogen peroxide [40]. Reduction in cellular frataxin in patients with FRDA is associated with free iron accumulation within the mitochondria, leading to increased levels of Fenton-reactive iron ions, and thus increasing cellular sensitivity to hydrogen peroxide mediated oxidative stress. Some studies suggest links between frataxin expression, oxidative stress and the impairment of the anti-oxidant enzymes glutathione peroxidase and catalase [44]–[48]. We therefore investigated the effect of human MSCs or *FXN*-transfection has on the expression of these intracellular hydrogen peroxide scavenging enzymes. Firstly it was shown that there was a deficiency in both catalase and glutathione peroxidase 1 in patients with FRDA when compared to healthy controls. Furthermore, *FXN*-transfection or human MSCs, via the secretion of soluble factors, are able to increase catalase and/or glutathione peroxidase 1 protein levels in fibroblasts derived from patients with FRDA. Exposure to MSC-conditioned media or *FXN*-transfection did not result in any significant increases in enzymes expression in healthy control cells. These results are therapeutically interesting as administration of enzymes that scavenge hydrogen peroxide, such as catalase, or the addition of hydrogen peroxide scavenging enzyme mimetics may normalize the antioxidant activity and suppress the deleterious phenotypes associated with frataxin deficiency [38], [49], [50].

We have confirmed observations made by others demonstrating that fibroblasts from healthy controls were more resistant to hydrogen peroxide-mediated oxidative stress compared to FRDA fibroblasts [35]–[38]. We have shown that prior incubation of FRDA fibroblasts with MSC-conditioned medium before hydrogen peroxide exposure results in an increase in resistance to hydrogen peroxide-mediated cell toxicity. MSCs are known to secrete a vast plethora of effector cytokines and growth factors known for their cellular protective properties, and within our laboratories we have previously demonstrated that MSCs protect cells against toxic insults via modulation of signalling cascades such as the PI_3_kinase/Akt and p38 MAPkinase pathways [17], [18]. Pre-exposure to MSC-conditioned media on the other hand did not result in rescue of fibroblasts from healthy controls. Increasing frataxin expression in FRDA fibroblasts using *FXN*-transfection, in addition to increasing catalase expression, also rescues cells from hydrogen peroxide exposure. Both the addition of exogenous catalase or glutathione peroxidase rescue FRDA fibroblasts against hydrogen peroxide toxicity. Furthermore, the presence of a catalase inhibitor abrogates the increase in FRDA fibroblast survival, caused by exposure to MSC-conditioned medium during insult by hydrogen peroxide. Overall these results suggest a direct link between frataxin, levels of catalase expression and sensitivity to hydrogen peroxide mediated toxicity. We hypothesize therefore that an increase in frataxin, catalase and/or glutathione peroxidase 1 levels in fibroblasts derived from patients with FRDA after exposure to MSC-conditioned medium results in, at least in part, this rescue effect. Therapeutically, MSCs may be able to rescue normal function and normalize antioxidant activity in frataxin defective cells by increasing frataxin levels above a specific threshold at which point patients' with FRDA become asymptomatic.

Currently there are no effective treatments in preventing the progression of FRDA. Data in this study demonstrates for the first time that MSCs secrete factors that increase cellular frataxin levels in frataxin-deficient fibroblasts and protect those cells from oxidative damage. Increasing evidence suggests a major protective role of MSCs is their capacity to secrete a diverse range of potentially trophic, paracrine and protective factors, in addition to anti-oxidant actions, thereby promoting an environment conducive to cellular protection and support diseased cell survival [15]–[20], [51], [52]. Transplantation of MSCs has the potential to be an effective treatment for FRDA through a multitude of different mechanisms. A number of studies have shown that intravenous delivery of bone marrow derived cells, in humans and experimental animals, results in significant numbers of cells entering the CNS parenchyma, including the cerebellum [21], [53]–[55]. Further to this, following transplantation of MSCs for both neurological and myocardial injury, significant improvements in many clinical parameters tested have been demonstrated [21], [56]–[58]. Administration of MSCs is an attractive therapeutic option in FRDA: MSCs are easily isolated from various anatomical sources, have a versatile growth and differentiation potential, and, by virtue of their immunosuppressive properties, have potential as either an allogeneic or autologous cell therapy. Furthermore the safety of injecting MSCs intravenously in humans has been extensively confirmed in a number of diseases [59]. Rapid clinical translation of our experimental studies could therefore be justified. Overall our findings demonstrate, for the first time, a potential role for cell-based therapies in FRDA and, in time, transplantation of bone marrow-derived MSCs may offer an effective treatment in these patients.
